# An Update on Recent Studies Focusing on the Antioxidant Properties of *Salvia* Species

**DOI:** 10.3390/antiox12122106

**Published:** 2023-12-13

**Authors:** Domenico Iacopetta, Jessica Ceramella, Domenica Scumaci, Alessia Catalano, Maria Stefania Sinicropi, Rosa Tundis, Stefano Alcaro, Fernanda Borges

**Affiliations:** 1 Department of Pharmacy, Health and Nutritional Sciences, University of Calabria, 87036 Arcavacata di Rende, Italy; domenico.iacopetta@unical.it (D.I.); jessica.ceramella@unical.it (J.C.); rosa.tundis@unical.it (R.T.); 2 Laboratory of Proteomics, Department of Experimental and Clinical Medicine, Magna Græcia University of Catanzaro, “S Venuta” Campus, 88100 Catanzaro, Italy; scumaci@unicz.it; 3 Research Center on Advanced Biochemistry and Molecular Biology, Magna Græcia University of Catanzaro, “S Venuta” Campus, 88100 Catanzaro, Italy; 4 Department of Pharmacy-Drug Sciences, University of Bari “Aldo Moro”, Via Orabona 4, 70126 Bari, Italy; 5 Department of Health Sciences, University “Magna Græcia” of Catanzaro, Viale Europa, 88100 Catanzaro, Italy; alcaro@unicz.it; 6 Net4Science SRL, Academic Spinoff, Università “Magna Græcia” di Catanzaro, Viale Europa, 88100 Catanzaro, Italy; 7 Associazione CRISEA-Centro di Ricerca e Servizi Avanzati per l’Innovazione Rurale, Loc. Condoleo, 88055 Belcastro, Italy; 8 CIQUP-IMS/Department of Chemistry and Biochemistry, Faculty of Sciences, University of Porto, Rua do Campo Alegre s/n, 4169-007 Porto, Portugal; fborges@fc.up.pt

**Keywords:** *Salvia* species, extraction methods, antioxidant assays, antioxidant properties

## Abstract

Nutrition has crucial effects and a significant role in disease prevention. Recently, nutraceuticals have attracted much attention in scientific research due to their pleiotropic effects and relatively non-toxic behavior. Among the biological effects displayed by plants belonging to the Lamiaceae family, such as antibacterial, anticancer, anti-inflammatory, and anticholinesterase, sage is well known for its antioxidant properties and is a rich source of numerous compounds that are biologically active, amongst them polyphenols, with more than 160 types identified. In this review we summarized some of the significant studies published in the last decade reporting the most employed extraction methods and the different assays that are useful for establishing the antioxidant properties of some sage species. Even though the scientific literature contains plenty of data regarding the antioxidant properties of many sage species, further studies are needed in order to gain a deeper understanding of the mechanism of action and the compounds responsible for their antioxidant activity. Finally, it should be taken into account that the data on the antioxidant properties of sage extracts are often difficult to compare with each other, since a series of variables in the extraction procedures, the type of assay used, and standardization may affect the final result.

## 1. Introduction

**Figure 1:**
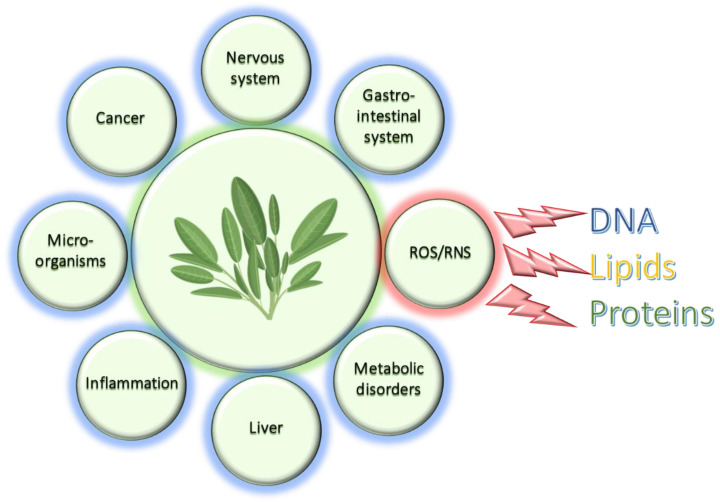
*Salvia* species extracts’ main benefits.

In the last decade, increased attention has been paid to healthier lifestyles and nutrition, with a net trend toward the consumption of foods and supplements rich in phytochemicals that may prevent different diseases such as cancer, diabetes, neurodegeneration, and cardiovascular system disorders, among others [1]. More recently, many studies have been focused on nutraceuticals derived from natural sources, such as plants, able to prevent and treat a wide range of pathologies and whose mechanisms are still under-investigated [2]. Amongst the several medicinal plants with beneficial effects on human health, sage species have attracted the attention of numerous researchers because of their multiple biological properties for preserving good health and treat different diseases [3,4]. The genus *Salvia* L. is commonly known as sage and includes the most common *Salvia officinalis* L. (Dalmatian sage), *Salvia lavandulaefolia*, *Salvia fruticosa*, *Salvia miltiorrhiza*, and others [5], and is represented by approximately one thousand species worldwide [6,7]. Several biological activities have been reported for sage extracts, such as antibacterial, anticancer, anticholinesterase, antinociceptive, hypoglycemic, hypolipidemic, liver-protective, antioxidant, etc. (Figure 1) [8,9,10,11,12]. Recently, the anti-radical activity of *S. officinalis* L. against uranium toxicity, with uranium being a highly radioactive toxic heavy metal, has been suggested [13]. It is known that the overproduction of free radicals, namely nitrogen- (RNS) or oxygen-derived (ROS), is harmful for humans and other living organisms, and that these free radicals possess high reactivity and a short life, because unpaired electron(s) may extract electron(s) from biological molecules, such as DNA, proteins, and lipids, for gaining stability. An overproduction of ROS can occur through environmental causes (pollution, cigarette smoke, ozone, and ultraviolet (UV) radiation, for instance) or endogenously under physiologic or pathologic conditions (amino acids oxidation, the mitochondrial electron transport chain, respiratory burst by phagocytes, ischemia–reperfusion injury, etc.). Normally, the presence of endogenous and exogenous antioxidants, for instance, from food intake, balances the produced RNS and ROS, but when this equilibrium is broken, the oxidative stress becomes harmful and may lead to several chronic diseases.

**Figure 2:**
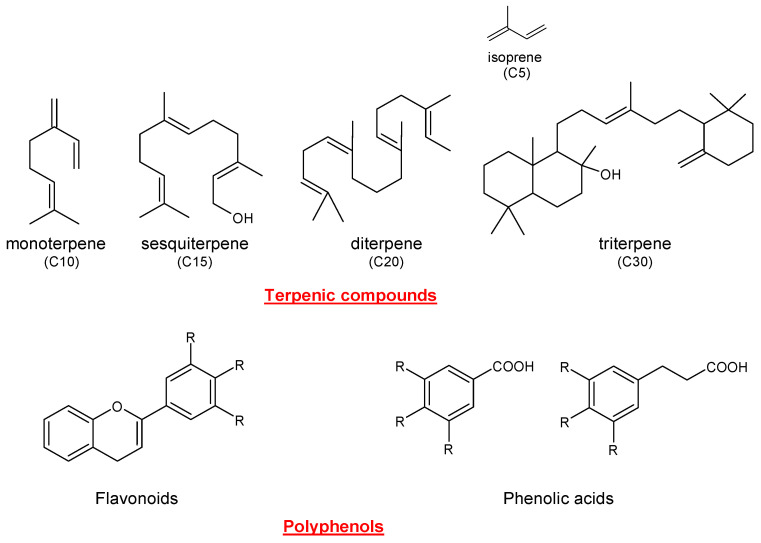
Representative structures of terpenic compounds and polyphenols.

Sage contains many biologically active compounds, including phenolic components [14] and monoterpenes, sesquiterpenes, diterpenes, and triterpenes, based on the isoprenic units contained in the structure (two units = monoterpene; three units = sesquiterpenes; four units = diterpene; six units = triterpene, Figure 2). Phenolic components can be roughly divided into two groups: flavonoids (luteolin, apigenin, and quercetin) and phenolic acids (caffeic, vanillic, ferulic, and rosmarinic acid) [15]. The most common terpenes present in sage include α- and β-thujone, 1,8-cineole, and camphor (monoterpenes); carnosic acid, carnosol, rosmanol, rosmadial, and manool (diterpenes); oleanolic and ursolic acids (triterpenes); along with α-humulene and viridiflorol (sesquiterpenes) [16]. Moreover, the presence of luteolin methyl carnosate, rosmadial, 9-ethylrosmanol ether, epirosmanol, isorosmanol, and galdosol has been described in extracts from *S. officinalis* and *S. fruticosa* [17,18,19]. Sage essential oil (EO) mainly contains α-thujone, camphor, viridiflorol, 1,8-cineole, and α-pinene and exerts antibacterial, antifungal, and free radical scavenging activity [20]. A growing number of studies support the modulation of neurotransmitter metabolism by *S. officinalis* extracts, which contribute to the improvement of cognitive performance in human volunteers [21,22,23]. However, even though the major components of the used extracts have been quantitatively and qualitatively characterized, their biological effects are attributed to the phyto-complex rather than to the single component. Moreover, particular importance has been recently attributed to *S. miltiorrhiza* for the treatment of coronary heart disease, hypertension, ischemic stroke, angina pectoris [24,25,26], and viral diseases, including COVID-19 [27,28]. These activities are likely related to the presence of quinone diterpenes, also known as tanshinones, including tanshinone I, tanshinone IIA, dihydrotanshinone I, cryptotanshinone, and hydroxytanshinone. Among these, the most interesting is tanshinone IIA, which demonstrated cardiovascular-protective [29,30] and renoprotective [31] activities, and antiviral activity [32]. It has also been recently investigated as a natural anticancer compound, due to its inhibitory effect on cancer with a certain regulatory effect on tumor angiogenesis [33,34,35]. Several recent papers have also addressed the large-scale production of *Salvia* spp. using efficient preservation processes [36,37,38,39,40,41]. All substances present in *Salvia* spp. have been widely studied for their diverse biological activities. In this review, our interest was focused on the antioxidant activity of several extracts of sage. The most common extraction methods, as well as several in vitro and in vivo studies regarding the antioxidant activity of diverse sage species (spp.), are herein reported.

## 2. Extraction Methods

Sage species have been widely used in popular medicine for their biological properties, and many different methods for the extraction and identification of these components have been reported [42]. Today, various techniques are used for obtaining various sage products [43] and are chosen depending on the desired profile of sage’s bioactive compounds in an extract, and the most used techniques have been recently extensively summarized [16]. Currently, the most employed extraction methods (Table 1) are represented by hydrodistillation (HD) [44], steam distillation (SD) [45], ultrasound-assisted extraction (UAE) [46], sonohydrodistillation (SHD) [47], microwave-assisted extraction (MAE) [48] including microwave-assisted hydrodistillation (MHD or MAHD) [49,50], solid–liquid extraction (SLE) [51], Soxhlet extraction (SE) [52], infusion [53], freeze drying (FD) [54,55], solvent-free microwave-assisted extraction (SFME) [56], supercritical fluid extraction (SFE) [57], subcritical water extraction (SCWE) [58], and supercritical CO_2_ extraction (SC-CO_2_) [59,60]. The choice of extraction technique was seen to influence phenolic acids and flavonoid composition, where ultrasound-assisted extraction (UAE) gave the highest concentration [61]. They will herein be briefly described, together with their pros and cons. HD is the most commonly used method to obtain sage products, mainly directed toward the production of EO, and it uses a Clevenger-type apparatus, with some modifications [62]. Although HD is an old and simple technique for EO extraction, at the industrial level, it has been replaced with steam distillation, the parameters of which have been modified in order to make it less expensive [63]. UAE is an efficient technique, with lower equipment costs and is used in large-scale applications. It is based on applying high-frequency sounds and a limited amount of solvent to achieve effective extraction of the components contained in a solid matrix [64]. Sonohydrodistillation is an innovative approach, as waves generated from sonication might make hydrodistillation more rapid by creating the physical amendments for improved mass and heat transfer [65]. MAE is a simple, low-cost, and modern extraction technique with a reduced extraction time and solvent employment that can process a high amount of raw material. A limitation is the extraction of volatile or thermo-sensitive components, because of the cooling or venting periods required after the extraction process [66]. Numerous studies of *S. officinalis* L. are carried out on the crude products obtained by solid–liquid extraction by using different solvents and comparing both classical and innovative extraction techniques. For instance, maceration is a simple and the most common form of solid–liquid extraction, in which a proper solvent is added to the crushed plant material and shaken. In the case of the industrial production of extracts, solvents are allowed to circulate through the plant material, and multiple extraction is often used [67]. Soxhlet extraction is another conventionally used method, but needs long extraction times and organic solvents, most of them toxic and flammable [68]. A very simple and widely used technique, generally employed for galenical preparations, especially in the past, is infusion that involves macerating the plant’s parts in boiling water for a short period of time. This technique produces a deposit because of the coagulation of the inert colloidal material. These kinds of extracts must be used within a few hours due to the high propensity of microbial growth and are not acceptable for large-scale production; however, if alcohol is added to the infusion, during or after the extraction process, the problem is over. This method has been successfully used in some studies [69]. FD, also known as lyophilization, is a well-known technique for the production of high-quality food powders and solids [70]. It is a preferred method for drying foods containing compounds that are thermally sensitive and prone to oxidation since it operates at low temperatures and under high vacuum. FD of food and biological materials has the advantage of minimal loss of flavor and aroma. It requires very low pressures or high-vacuum conditions to produce a satisfactory drying rate [71]. SFME is an efficient and eco-friendly technique, where the operational aspects of MAE have been maneuvered to make it compatible with the extraction of EOs [72]. SFE has been highlighted in the literature, thanks to its advantages related to the protection of photosensitivity, oxidizability, and volatility of biocompounds. It was also successfully used for the extraction of pigments and aromatic compounds, including alkaloids, from flowers, which are the most fragile plant organ and may contain a vast range of variable compounds [73]. SCWE is considered a safe, fast, economical, and environmentally friendly method, in which the use of water, subjected to high pressure, is needed to increase its temperature to above its normal boiling point. The use of water as the solvent for the extraction of EO is both cost-effective and environmentally friendly. Moreover, this technique requires significantly reduced extraction times (around 2–3 times), and the consumption of a lower amount of raw material, to produce a higher quality and quantity of EO [58]. Finally, SC-CO_2_ represents a promising and advantageous technology, with a dissolving ability comparable to organic solvents but with better diffusion, fast extract/solvent separation, and the possibility to recycle the supercritical fluid, and has been successfully used for the extraction of thermolabile components [74]. CO_2_ is an optimal solvent because it is natural, quite inexpensive, non-toxic and chemically inert, non-flammable, easily to remove, odorless, and flavorless. The use of SC-CO_2_ at high pressure has been demonstrated to be good method for the extraction of vegetable oils [75]. Even though CO_2_ is optimal for non-polar or slightly polar compounds, it has a low affinity for polar components, an inconvenience that can be overcome by adding polar co-solvents [76]. While CO_2_ is not expensive, the necessary equipment and the extraction process are, especially at higher pressures and temperatures; however, several approaches, for instance, maintaining the variable stream circulation of the solvent, have been proposed to surpass these drawbacks [77].

## 3. Methods for Evaluation of Antioxidant Activity

**Figure 3:**
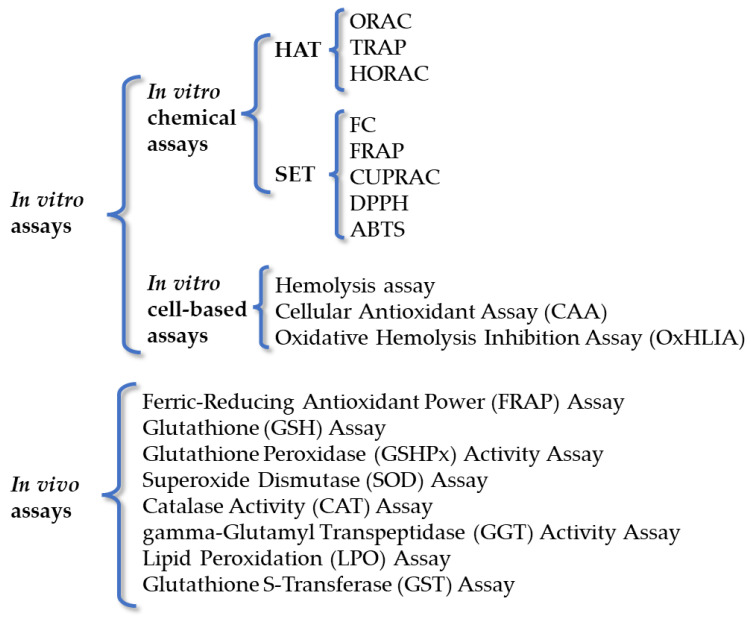
Schematic representation of the most employed antioxidant activity determination methods.

The evaluation of antioxidant activity has notably evolved in the past decade; indeed, early methods based on measuring lipid oxidation have been replaced with chemical tests coupled with innovative detection technologies. A direct measure of the transfer of hydrogen atoms or electrons from antioxidants to free radicals, coupled with their ability to neutralize radical species, may provide information on their intrinsic antioxidant potential and generally adopts a chemical system composed of an oxidant (ROS or other), an oxidizing compound, and the antioxidants to be studied [78]. Moreover, the method for determining antioxidant activity should be simple, reproducible, able to analyze hydrophilic and lipophilic antioxidants, appropriate for a determined in vitro or in vivo experiment, based on chemically defined reaction(s) and have and endpoint, and record a radical that is biologically relevant [79]. The available methods for antioxidant capacity evaluation are generally based on electrochemistry, spectrometry, and chromatography. They are briefly summarized in Figure 3.

### 3.1. In Vitro Chemical Assays

In vitro chemical assays can be divided into hydrogen atom transfer (HAT) and single electron transfer (SET) methods, on the basis of the chemical reactions possessing different kinetics and intermediates, but with the same final result for both [67]. Specifically, HAT methods measure the ability of an antioxidant to quench free radicals by hydrogen donation, whereas SET ones detect the ability of a potential antioxidant to transfer one electron and reduce any compound, including metals, carbonyls, and radicals. These tests are fast and can be automated and used for the initial screening of several antioxidants. Furthermore, they can be used as single or combined assays. HAT assays determine the ability of an antioxidant to remove free radicals through a hydrogen atom donation. Some examples are the Oxygen Radical Absorption Capacity (ORAC), the Hydroxyl Radical Antioxidant Capacity (HORAC), the Total Peroxyl Radical-Trapping Antioxidant Parameter (TRAP), and β-carotene bleaching assays [80,81,82,83,84,85,86].

Electron transfer (ET) tests, based on SET, detect the ability of an antioxidant to reduce metallic ions, carbonyl groups, and free radicals by transferring an electron, and are pH-dependent [87]. The Folin–Ciocalteu (FC), Ferric Reduction of Antioxidant Power (FRAP), and cupric reducing antioxidant capacity (CUPRAC) tests are included amongst these methods. The well-known FC test is widely used to measure the total phenolic content (TPC) in plant extracts and other biological samples, originally used to detect proteins, and then, developed to determine the antioxidant ability of different extracts [88,89]. Additionally, the DPPH (2,2-di(4-*tert*-octylphenyl)-1-picrylhydrazyl) and ABTS (2,2′-azinobis-(3-ethyl-benzothiazoline-6-sulfonic acid)) assays are the most common ones [69,90,91].

Finally, several authors have used mixed tests which are based on mixed mechanisms (HAT/SET). Briefly, they involve the elimination of a stable chromophore where HAT, ET, and proton-coupled electron transfer (PCET) mechanisms play different roles, depending on the pH, solvent, and other reaction conditions [87]. The main mixed assays are the ABTS/Trolox equivalent antioxidant capacity (TEAC), DPPH, and *N*,*N*-dimethyl-*p*-phenylenediamine dihydrochloride (DMPD) ones. The ABTS/TEAC assay is an easy and convenient test for measuring the total antioxidant capacity (TAC) of a pure compound, or extract, by measuring its ability to neutralize the ABTS stable radical cation [92,93].

### 3.2. In Vitro Cell-Based Assays

The evaluation of the antioxidant activity of several synthetic bioactive compounds and natural extracts has been conducted using in vitro cell-based assays, taking into account different variables such as cellular absorption, metabolism, and the cell’s environmental context. Cell models are ideal for developing a better understanding of the antioxidant activity nearer to that exerted in vivo, which is not always easy to measure directly using animal or human subjects. One of these tests is represented by the hemolysis inhibition assay, which is suitable for the determination of both hydrophilic and lipophilic antioxidants [94,95]. The cellular antioxidant assay (CAA) was also developed for the quantitative measurement of antioxidants’ ability to inhibit oxidation using a determined cell model [96,97]. Through this assay, it is possible to evaluate antioxidant capability under physiological conditions, and the cellular uptake of antioxidants can be correlated with bioavailability in in vivo systems, and it has been used for the evaluation of several compounds and extracts [98,99,100]. Finally, the oxidative hemolysis inhibition assay (OxHLIA) is based on the inhibition of free radical-induced membrane damage in erythrocytes by antioxidants. In this assay, the temperature-dependent free radical initiator AAPH is responsible for the formation of free (peroxyl) radicals, in the in vitro system, which attack the erythrocyte membranes and eventually cause hemolysis [101]. Since the peroxyl radicals formed in the in vitro system are also found in the human body, this cell-based assay has been pointed out as suitable for assessing the antioxidant activity of natural extracts. This method uses peroxyl radicals as pro-oxidants and erythrocytes as oxidizable targets, so that the results reflect the biologically relevant radical-scavenging activity and the micro-localization of antioxidants [94,102].

### 3.3. In Vivo Assays

Several in vivo animal studies have been performed to evaluate the antioxidant activity of natural extracts. Usually, oral or intravenous administration to the animals (mice or rats) at a definite dosage is used and, after a defined period, the animals are sacrificed, and blood or tissues are used for the evaluation of antioxidant activity using specific assays. The ferric reducing ability test is a rapid and useful routine test that estimates the antioxidant activity of a given extract/compound from animals’ blood samples, by using the FRAP reagent [103]. This assay gives the antioxidant index potential of biological fluids and is highly reproducible. The levels of reduced glutathione (GSH) can also be exploited for antioxidant activity determination [86]. Glutathione peroxidase (GSHPx) is a seleno-enzyme present in the cytosol and mitochondria that catalyzes the reaction of hydroperoxides with reduced GSH, forming glutathione disulfide (GSSG) and the reduction product of hydroperoxide. Antioxidant activity is detected by monitoring the conversion of NADPH to NADP^+^ [104]. Another assay exploits the following enzymes: glutathione-S-transferase (GSt) [105], superoxide dismutase (SOD) [106], catalase (CAT) [86], *gamma*-glutamyl transpeptidase ac (gGT) [106], and glutathione reductase (GR) [107]. Lipid peroxidation (LPO) is an autocatalytic process that produces malondialdehyde (MDA) as one of the end products. The peroxidation level is expressed as nanomoles of thiobarbituric acid (TBA)-reactive substances (TBARS)/mg protein [10,108]. As discussed, the antioxidant activity of the various sage extracts can be evaluated through different methods, both in vitro and in vivo. It is evident that the in vitro methods are the most frequently adopted and, amongst them all, DPPH, HORAC, and SOD are the most preferred by the scientific community. Additionally, amongst the in vivo assays, LPO, CAT, and GSHPx exhibit the highest frequency of use.

## 4. *Salvia* spp. Extracts and the Evaluation of Antioxidant Activity

The scientific literature reports a myriad of studies regarding the biological activities of several *Salvia* spp., including the most investigated antioxidant ones. It is commonly accepted that secondary metabolites, such as phenolic acids, flavonoids, and terpenes, are responsible for antioxidant activity, and different methods have been developed, as discussed above. Herein some significative examples taken from studies, published in the last decade, will be reported.

### 4.1. Antioxidant Activities of S. officinalis L. spp. Individually

Hamrouni-Sellami et al., (2013) [109] studied the influence of different drying methods on phenolics’ and flavonoids’ qualitative and quantitative content and the antioxidant activity of methanol extract from *S. officinalis* L. aerial parts (Table 2). The authors found out that the two drying techniques allowed for the improvement in antioxidant activity, assessed by DPPH and β-carotene bleaching assays, namely microwave (output power from 600 to 800 W) and far-infrared drying at 65 ⁰C. Fischedick et al., (2013) [110] isolated some phenolic diterpenes from an acetone extract of *S. officinalis* L. dried aerial parts, namely carnosic acid, carnosol, epirosmanol, rosmanol, 12-methoxy-carnosic acid, sageone, and carnosaldehyde, using hexane soluble material over a polyamide column, followed by centrifugal partition chromatography, and reverse-phased semi-preparative HPLC. Isolated compounds were identified by ^1^H-NMR, 2DCOSY, and LC-MS, and then, tested for their ability to regulate antioxidant and cytoprotective gene expression mediated by Nrf2 through quantitative PCR (qPCR). Their outcomes indicated that almost all the isolated compounds activated Nrf2-mediated gene expression in mouse primary cortical cultures and that, in particular, carnosol and carnosaldehyde were able to protect the cultures from H_2_O_2_ and oxidative stressors used to induce cell death. Martins et al., (2015) [111] prepared aqueous (infusion and decoction) and methanol/water (80:20, *v*/*v*) extracts of *S. officinalis* L. flowering aerial parts, and tested their antioxidant and antifungal activities, identifying some bioactive molecules, mainly phenolics (rosmarinic acid derivatives) and flavonoids (luteolin derivatives). Four different in vitro assays were performed: DPPH, reducing power, the inhibition of β-carotene bleaching, and lipid peroxidation inhibition. The best antioxidant properties were exhibited by the methanol/water extract, followed by the aqueous extracts obtained by decoction and infusion. Smach et al., (2015) [112] proved that the administration in mice of an aqueous extract of *S. officinalis* L. aerial parts produced antioxidant effects and inhibited acetylcholinesterase activity in the brain. These results suggest an important role of the extract in the prevention and amelioration of neurodegenerative disease symptoms. Antioxidant ability was measured with the DPPH scavenging assay (IC_50_ = 14.5 µg/mL), and the detected GSH and ascorbic acid levels in mouse brains were higher than in the control group. Reis et al., (2016) [113] prepared solid lipid nanoparticles (NPs), made of Witepsol and Carnauba waxes, loaded with rosmarinic acid, obtained from leaves of sage (*S. officinalis* L.) and savory (*Satureja montana*), and studied their antioxidant and safety profiles using in vitro and in vivo approaches. The NPs loaded with rosmarinic acid (0.15 mg/mL) exerted antioxidant/protective effects on the damage to DNA and reduced lipid peroxidation in rats. These results highlight the effectiveness and safety of NPs in protecting rosmarinic acid from gastrointestinal degradation and enhancing its bioavailability. Pavlić et al., (2016) [114] produced an extract from *S. officinalis* L. herbal dust, discarded as a by-product from filter tea, using the SCWE method, performed in a batch-type high-pressure extractor. In this way, total phenol (TP) and total flavonoid (TF) yields, together with antioxidant activity, determined by DPPH, ABTS, and a reducing power assay, were notably improved compared to the extraction by maceration. The authors propose that this technique can be applied for the smart reuse of this by-product for obtaining valuable bioactive compounds. Cutillas et al., (2017) [115] described the composition of *S. officinalis* L. subsp. *Lavandulifolia* (Vahl) Gams or Spanish sage EOs, obtained using the HD technique, by fast gas chromatography, with high percentages of camphor (30.8–37.2%), 1,8-cineole (21.7–25.7%), camphene (7.2–9.4%), α-pinene (4.8–5.5%), β-pinene (4.0–5.6%), limonene (2.8–4.4%), myrcene (1.3–1.6%), and sabinene (1.3–1.8%). The extensive enantiomeric distribution of EO components, such as sabinene hydrate, camphor, bornyl acetate, and borneol, was obtained in an enantioselective gas chromatography–mass spectrometry (EsGC-MS) study; then, the antioxidant activity was measured using ORAC, DPPH, ABTS, and reducing power methods. All the tested oils possessed noteworthy antioxidant activity, albeit with some differences between the diverse types of oil, mainly due to their individual composition and, obviously, to the adopted test. Pavić et al., (2019) [116] obtained carnosol and carnosic acid from *S. officinalis* L. leaves using SFE and demonstrated that only the adopted pressures (ranging from 10 to 30 MPa) significantly affected carnosol extraction, whereas pressure, temperature, and CO_2_ flow rate together significantly affected the amount of carnosic acid. The antioxidant activity was evaluated by the DPPH assay, and the extract obtained at 30 MPa and 40 °C with a 2 kg h^−1^ CO_2_ flow rate, a carnosic acid content of 72 µg mg^−1^, and a carnosol content of 55 µg mg^−1^ showed the highest antioxidant activity, at a concentration of 25 µg mg^−1^. Salević et al., (2019) [117] developed poly(ε-caprolactone) (PCL) films, loaded with a solid dispersion obtained from the maceration (water/ethanol 50% *v*/*v*) of *S. officinalis* L., through an electrospinning technique and annealing treatment. The authors prepared three PLC-loaded films, with sage contents equal to 5%, 10%, and 20%, and evaluated the physicochemical and functional properties of the films, together with some biological properties, namely the antioxidant profile, finding that in the PCL-based films there was an almost two-fold increase in the antioxidant power evaluated by the DPPH assay, suggesting potential employment of this type of system in food products. Tundis et al., (2020) [118] obtained three different EOs from fresh aerial parts *S. officinalis* L. by HD using a Clevenger-type apparatus. The herbal parts were harvested from Calabria (Italy) and characterized qualitatively and quantitatively by gas chromatography (GC) and gas chromatography–mass spectrometry (GC-MS), revealing that the oxygenated monoterpenes, particularly camphor and 1,8 cineole, were the most represented. The antioxidant capacity of EOs was evaluated in vitro by means of DPPH, ABTS, FRAP, and β-carotene bleaching assays, which, together with the potential inhibitory activity against AChE and BChE enzymes, makes these extracts potentially useful for neurodegenerative disorders management. The aim of Siakavella et al., (2020)’s [119] work was to prepare silver nanoparticles (AgNPs), using green chemistry, and hydroglycolic extracts of medicinal plants, amongst them *S. officinalis* L. These NPs were mainly spheric and possessed good antioxidant activity, due to the phenolic and flavonoid content of their surface. The antioxidant activity was determined using the DPPH method. AgNPs showed strong antioxidant activity (IC_50_ = 0.77 ± 0.04 mg/mL) compared to the extract of sage (15.05 ± 0.49 mg/mL), almost comparable to that of ascorbic acid (0.24 ± 0.00 mg/mL). Francik et al., (2020) [69] prepared methanol–acetone extracts and infusions from *S. officinalis* L. variety *Bona* leaves, collected during the plant’s blossoming period (June and July) and dried naturally or at 35 °C. The antioxidant activity was assayed in methanol–acetone extracts and infusions of dried leaves with the DPPH and FRAP assays. The natural drying conditions allowed for better antioxidant activity than drying at 35 °C; however, the authors indicated that the July harvest, regardless of the drying method, possesses the best antioxidant activity, together with a higher presence of polyphenolic compounds. In both extracts and infusions from the leaves dried at 35 °C, 3,5-dicaffeoylquinic acid, sinapinic acid, *p*-coumaric acid, isorhamnetin, and catechin were present in the same amounts, whereas ferulic acid, hesperidin, and rutin were found in higher amounts in naturally dried leaf extracts. Thus, the authors suggested that the methanol–acetone extracts and infusions of dried leaves from *S. officinalis* L. (variety *Bona*) had different antioxidant capacities related to the harvesting time and drying method. Jedidi et al., (2020) [86] investigated the individual and synergistic protective properties of *S. officinalis* L. flower decoction extract and sulfasalazine in a rat model of an ethanol-induced peptic ulcer. The dried flowers were powdered and extracted by decoction with distilled water (1/5; *w/v*) at 100 °C. The antioxidant activity was tested in vitro by the β-carotene bleaching inhibition assay, with an IC_50_ of 56.77 ± 2.34 µg mL^−1^, and in vivo, measuring SOD, CAT, and GSHPx activities in the stomach and intestinal mucosa. The loss of acute EtOH-induced oxidative stress was due to the high levels of phenolic acids, flavonoids, and polyols, such as quinic, protocatechuic, 1,3-di-*O*-caffeoyquinic, *p*-coumaric, and salviolinic acids, and naringin, quercetin, kampherol, apigenin-7-*O*-glucoside, luteolin-7-*O*-glucoside, and cirsilineol, present in the extract, and was increased in the co-treatment with sulfasalazine. Ueda et al., (2021) [120] developed an enriched extract from dried leaves of *S. officinalis* L., using optimized ultrasound-assisted extraction (UAE), with the aim of boosting their antioxidant and antimicrobial properties to be exploited as natural preservatives in yogurts. By determining antioxidant activity through OxHLIA, IC_50_ values were calculated for time periods of 120 and 180 min, i.e., the extract concentration required to protect 50% of the erythrocyte population from the hemolytic action of AAPH for 120 and 180 min (2.6 ± 0.2 µg/mL and 8.8 ± 0.4 µg/mL, respectively) compared to Trolox (41 ± 1 µg/mL and 63 ± 1 µg/mL, respectively). Moreover, sage extract was demonstrated to be not hepatotoxic. Cvitković et al., (2021) [121] studied the chlorophyll and carotenoid profiles in the extracts obtained from leaves of various plants, including *S. officinalis* L., using successive extraction with three solvents of different polarities (hexane, acetone 80%, and ethanol 96%). The antioxidant capacity, determined by the FRAP method, was found to be high for *S. officinalis* L., and it was related to the levels of lutein, β-carotene, zeaxanthin, 9-cis lutein, and chlorophyll *b* in *S. officinalis* L. extracts. Đurović et al., (2022) [122] investigated the effects of the preparation procedure on the chemical composition, thermal behavior, and antioxidant activity of EOs extracted through classical HD and MHD from *S. officinalis* L. leaves. They found out that, in all samples, viridiflorol was the principal compound, followed by 1,8-cineole (eucalyptol), α-and β-thujones, camphor, borneol, and verticiol, whereas the concentrations of minor compounds were significantly different. The antioxidant activity was determined by DPPH, CUPRAC, FRAP, ABTS, HRSA, and TBARS and α-thujone and menthone content was related to the most positive effect in the first five assays, whereas verticiol and valencene had a negative influence. Finally, α-thujone, menthone, camphor, and carvyl acetate positively influenced the TBARS assay. Jedidi et al., (2022) [123] evaluated the antioxidant properties of *S. officinalis* L. flower aqueous extract. By means of the HPLC-PDA/ESI-MS method, four phenolic acids, including quinic acid, protocatechuic acid, 1,3-di-O-caffeoyquinic acid, and *p*-coumaric acid, and eight flavonoid compounds, amongst which the main ones were trans-cinnamic acid, catechin (+), naringin, and quercetin, were identified. The presence of these compounds has been related to their strong ABTS scavenging ability (IC_50_ = 52.58 ± 4.13 μg/mL) and to their in vivo protective effect against oxidative stress in rats. Indeed, *S. officinalis* L. flower aqueous extract treatment diminished the depletion of SOD, CAT, and GPx enzymatic activities, counteracting lipoperoxidation and, overall, protecting the gastrointestinal tract from inflammation and peptic ulcers. The study of Hrebień-Filisińska and Bartkowiak (2022) [124] assessed the quality of a macerate obtained from *S. officinalis* L., variety *Bona*, and fish oil that extended the shelf life of fish oil and is characterized by the presence of polyphenols, particularly carnosic acid, and plant pigments. This natural “green” macerate possesses good antioxidant properties and is safe; thus, it could also be used for the preservation of other food products. Mot et al., (2022) [125] analyzed, by GC-MS, three samples of *S. officinalis* L. EOs indicating the presence of 1,8-cineole, thujones, borneol, camphor, sabinene, camphene, and caryophyllenes as the principal components. Even though the antioxidant capacity determined by DPPH and ABTS assays was low (33.61% and 84.50% inhibition, respectively), the authors suggest the use of EO with a high borneol content in aromatherapy for hospitalized patients.

### 4.2. Antioxidant Activities of S. officinalis L. and Other Species (S. elegans, S. greggii, S. sclarea, S. hispanica, S. africana, and S. mexicana)

Pereira et al., (2018) [126] investigated the phenolic profiles and antioxidant activity of decoctions from three *Salvia* species, namely *Salvia elegans* Vahl., *Salvia greggii* A. Gray, and *S. officinalis* L. (Table 3). The *S. elegans* decoction was the most active, as demonstrated by the DPPH assay, with an EC_50_ of 10.7 ± 2.1 µg/mL, and its ability to reduce Fe^3+^, with an EC_50_ of 31.3 ± 5.0 µg/mL, and was correlated with a high concentration of caffeic acid and its derivatives, whereas the *S. officinalis* L. decoction inhibited xanthine oxidase activity, because of its richness in flavones, such as the glycosidic forms of apigenin, scutellarein, and luteolin. Afonso et al., (2019) [127] explored the phenolic composition and the antioxidant, anti-inflammatory, cytotoxic, and antibacterial activities of aqueous extracts of *S. africana*, *S. officinalis ‘Icterina’*, and *S. mexicana*, which are not commonly studied cultivars. Rosmarinic acid was the main phenolic compound in all extracts, but 40% of total phenolics was represented by yunnaneic acid isomers in *S. africana*, whereas *S. officinalis ‘Icterina’* extract included the apigenin, luteolin, and scuttelarein glycosidic forms. High antioxidant activity was exerted by the aqueous extract of *S. africana*, as determined by the DPPH, iron-reducing power, inhibition of β-carotene bleaching, and TBARS assays. Ovidi et al., (2021) [92] reported the liquid- and vapor-phase chemical composition, investigated by the GC-MS and HS-GC/MS techniques, of *S. sclarea* and *S. officinalis* EOs and hydrolates (HYs) from Tuscany (Italy). The antioxidant activity was assessed by DPPH and ABTS assays, together with an analysis of antibacterial activity by microdilution and the disc diffusion method. 1,8-cineole was the most abundant molecule in the EO liquid and vapor phases (30.4% and 48.4%, respectively) and HYs (61.4%) of *S. officinalis* L. Linalyl acetate was the main molecule detected in *S. sclarea* EOs (62.6% and 30.1% in liquid and vapor phases, respectively), whereas linalool was majorly present in HY (89.5%). The work by Gad et al., (2022) [128] reported the chemical profiles and the antioxidant activities of EOs extracted from the aerial parts of *S. officinalis* L., *S. virgata*, and *S. sclarea*. The samples were air-dried in the shade and EOs were hydro-distilled using Clevenger-type apparatus; the antioxidant activity of the EOs was evaluated using six in vitro assays. The *S. virgata* EO showed moderate antioxidant activity in the DPPH, ABTS, CUPRAC, and FRAP assays in comparison with the other two EOs. The major identified compounds were *cis*-thujone, 2,4-hexadienal, and 9-octadecenoic acid in *S. officinalis* L., *S. virgata*, *and S. sclarea* EOs, respectively. The principal component analysis (PCA) score plot suggested significant discrimination of the three species, without identifying the responsible compounds, as supported, as well, by the hierarchical cluster analysis. Dziadek et al., (2022) [129] investigated how different drying methods and periods of storage affected the antioxidant properties of Chia (*S. hispanica* L.), in comparison with *S. officinalis* L. and *S. sclarea* L. The fresh Chia methanolic extract possessed antioxidant activity of 713.26 ± 36.72 µmol Trolox g^−1^ of dry weight, determined using the ABTS method, which increased when the extract underwent freeze-drying to 1069.05 ± 33.52 µmol Trolox g^−1^ of dry weight. Amongst the different drying methods (freeze-drying, natural drying, and drying at 30, 40, and 50 °C), freeze-drying allowed for the best preservation of polyphenols and carotenoids. *S. hispanica* L. was found to be rich in rosmarinic acid, sinapinic acid, naringin, rutin, and carnosol, and storage up to 12 months reduced this content and, consequently, antioxidant activity.

### 4.3. Antioxidant Activities of S. miltiorrhiza, S. verbenaca, S. chamelaeagnea, S. bulleyana, S. multicaulis, and S. glutinosa

Fei et al., (2013) [130] reported that salvianolate, a water-soluble compound from *S. miltiorrhiza* Bunge, inhibited ROS and NOS production in H_2_O_2_-treated mouse cardiomyocytes through the downregulation of Smad2/3 and TGFβ1 expression. This effect was dose-dependent, but at high concentrations (5 g/L), salvianolate exhibited cytotoxicity in cardiomyocytes. Liu et al., (2014) [131] isolated two stereoisomers, (*R*)-norsalvianolic L and (*S*)-norsalvianolic acid L, from *S. miltiorrhizae* radix and rhizoma lyophilized powder. The powder was first dissolved in water, and then, subjected to AB-8 macroporous resin and polyamide column chromatography, followed by Sephadex LH-20 and ODS column purification. Finally, the compounds were obtained by preparative HPLC. These isomers were chemically characterized by different methods (such as 1D and 2D NMR (1H-1H COSY, HSQC, and HMBC) and circular dichroism experiments) and tested for their antioxidant properties using DPPH and ABTS microplates, giving IC_50_ values for (*R*)-norsalvianolic acid L of 6.9 and 9.7 μM and for (*S*)-norsalvianolic acid L of 27.1 and 25.3 μM, respectively. Belkhiri et al., (2017) [94] investigated different biological properties of *S. verbenaca* L. aerial part extracts (SVEs) harvested in the East of Algeria (during the period of April–May, at the flowering stage). The authors used different solvent extraction methods and, finally, they obtained an organic phase (ethyl acetate extract), which was the richest in polyphenols and flavonoids, and an aqueous fraction. As expected, the obtained fractions possessed antioxidant properties, mostly the organic fraction, as demonstrated using different assays, namely inhibition of AAPH-inducing erythrocyte hemolysis and chemicals-based assays, such as the reducing power, DPPH free radical, and ferrous ion-chelating activity ones. They consequently concluded that the potent antioxidant properties (for instance, with an IC_50_ of 0.0086 mg/mL for ethyl acetate extract in the DPPH assay) may be due to the presence of phenolics, flavonoids, tannins, etc., in the examined extracts. Zhang et al., (2018) [132] examined 50 batches of Chinese *S. miltiorrhiza* dried root powder, extracted with 70% methanol and ultrasonication, using ultra-performance liquid chromatography coupled with triple quadruple mass spectrometry (UPLC-Qqq-MS/MS). The use of a multivariate, statistical approach, PCA and bivariate correlation analysis, together with DPPH and ABTS assays, allowed us to understand the correlation between the identified secondary metabolites, mostly phenolic acids and tanshinones, with the antioxidant activities of the extracts. Etsassala et al., (2019) [133] reported the in vitro antioxidant activity of five terpenoids and one flavonoid compound (carnosol, carnosic acid, 7-ethoxyrosmanol, ursolic acid, rosmanol, and ladanein) purified through different techniques, including semi-Prep-HPLC, from a methanolic extract of *S. chamelaeagnea* leaves from South Africa. Strong antioxidant activity was recorded for carnosol and rosmanol by means of TEAC, ORAC, FRAP, and inhibition of Fe^2+^-induced lipid peroxidation assays, which mainly depends on the -OH groups, conjugation, and lactone ring present in these molecules. Grzegorczyk-Karolak et al., (2020) [134] determined the phytochemical profile of hydromethanolic extracts obtained from *S. bulleyana* aerial and underground parts, for the first time. The antioxidant activity of the extracts was studied by FRAP, free radical scavenging, and inhibition of lipid peroxidation assays. Even though the total content of phenolic compounds was higher in the roots than in the aerial parts, and the two extracts exhibited similar antioxidant activity, suggesting that the flavonoids, found only in the aerial part, gave the high contribution. Rowshan et al., (2020) [135] reported a study on the aerial parts of *S. multicaulis*, which contain high amounts of rosmarinic acid, catechin, vanillin, chlorogenic acid, quercetin, and *p*-coumaric acid, and possess good antioxidant activity, equal to 8.44 mg/g, as demonstrated by the DPPH scavenging assay, and with a content of phenol of 4.39 mg/g of the dried plant. Nicolescu et al., (2022) [53] reported some interesting results on an unusual species of *Salvia*, namely *S. glutinosa* L., the stems and leaves of which were harvested in two different locations in Romania. Two types of extracts, infusion (water extraction with heat) and maceration (hydroalcoholic extraction, room temperature, in the dark), were obtained, characterized qualitatively and quantitatively by LC-DAD-ESI/MS and investigated for their antioxidant properties by means of DPPH, ABTS, and FRAP assays. The phytochemical analysis found a polyphenol composition, rich in rosmarinic acid, luteolin acetyl-glucoside, and some types of *O*-hexosides. The antioxidant capacity of these extracts was evaluated in vitro, using DPPH, ABTS, and FRAP assays, and in vivo, through the assessment of some oxidative stress biomarkers, such as malondialdehyde (MDA), total thiols (SH), and total serum nitrates and nitrates (NOx), also useful for the determination of anti-inflammatory ability in a rodent model. The hydroalcoholic extracts showed higher activity compared to the infusions, and the prophylactic administration of the extract induced an increase in antioxidant levels in rat serum, which was associated with the anti-inflammatory effect.

## 5. Conclusions

Sage is a plant of considerable interest, given its high potential from a nutritional and biological point of view. Its usefulness in various diseases is widely reported by an ever increasing number of scientific publications. Among them, the interesting and well-documented antioxidant properties of this plant have been herein highlighted and discussed in detail. Fortunately, scientists utilize different methods for determining antioxidant properties, making their choice on the basis of the extract or phyto-complex to be studied. However, this variety of tests could represent, at the same time, one of the most debated questions, since it is very hard to compare the various methods even for a given extract. Moreover, the richness in the obtained and published data could be dispersive for the reader, and the number of variables produces, in most cases, very different results. Thus, it would be desirable to find a way to select and standardize the method used for recording and reporting the obtained outcomes. It would also be advantageous for the extracts to be qualitatively and quantitatively characterized, for the possibility of synergy and/or antagonism amongst the contained compounds to be considered, and to undertake the systematic reorganization of specific/thematic existing databases. Moreover, most of the studies are limited to in vitro or animal ones, lacking an adequate number of reports in humans. More recently, a trend toward pre-clinical and clinical studies that are focused on the effects of various *Salvia* species extracts on cognitive performance has been recorded. However, the encouraging results obtained are affected by some factors, such as the small number of participants, the lack of a pharmacopoeia standardization, and the (short) length of observation periods. Additionally, most of the bioactive components possessing interesting in vitro antioxidant activities could fail in human studies, since other parameters, such as bioavailability, intestinal permeability, and liver metabolization, may play a fundamental role. Finally, is necessary to continue with studies to delve deeper into the mechanism of action and assess the components responsible for its numerous activities. It must be also considered that the different species of sage possess very variable composition, influenced, for instance, by the time and place of harvesting, the soil and microclimate, etc. From this point of view, a comparison of multiple studies is necessary, in order to allow for easier tracing of the essential compounds responsible for the different activities. Finally, it is essential to develop improved knowledge about the bioactive potential of plant metabolites, aiming for the desirable development of new functional foods, nutraceuticals, and drugs based on plants.

## Figures and Tables

**Table 1:** Methods of extraction commonly used for sage species.

Method of Extraction	Acronym	Refs.
Hydrodistillation	HD	Aćimović et al., 2022 [44]
Steam Distillation	SD	Machado et al., 2022 [45]
Ultrasound-Assisted Extraction	UAE	Moussa et al., 2022 [46]
Sonohydrodistillation	SHD	Benmoussa et al., 2023 [47]
Microwave-Assisted Extraction	MAE	Peng et al., 2022 [48]
Microwave-Assisted Hydrodistillation	MHD or MAHD	Mohamed et al., 2022 [49]
Solid–Liquid Extraction	SLE	Didion et al., 2022 [51]
Soxhlet Extraction	SE	Vieira et al., 2020 [52]
Infusion	-	Nicolescu et al., 2022 [53]
Freeze-Drying	FD	Mondor et al., 2023 [54] Wang et al., 2022 [55]
Solvent-Free Microwave-Assisted Extraction	SFME	Liu et al., 2022 [56]
Supercritical Fluid Extraction	SFE	Huang et al., 2012 [57]
Subcritical Water Extraction	SCWE	Samadi et al., 2020 [58]
Supercritical CO_2_ Extraction	SC-CO_2_	Fikri et al., 2022 [59] Alara et al., 2021 [60]

**Table 2:** Antioxidant activity of *S. officinalis* L. coming from different countries.

Species	Material	Country	Extract	Antioxidant Activity Determination Method	Refs.
*S. officinalis* L.	aerial parts	Tunisia	methanol extract	DPPH, β-carotene bleaching	Hamrouni-Sellami et al., 2013 [109]
*S. officinalis* L.	dried aerial parts	Netherlands	acetone extract	CAA	Fischedick et al., 2013 [110]
*S. officinalis* L.	flowering aerial parts	Spain	methanol/water (80:20, *v*/*v*) extract	DPPH, β-carotene bleaching, lipid peroxidation inhibition	Martins et al., 2015 [111]
*S. officinalis* L.	aerial parts	Tunisia	aqueous extract	DPPH, GSH	Smach et al., 2015 [112]
*S. officinalis* L. and savory (*Satureja montana*)	leaves	Portugal	solid-lipid NP aqueous extract	TBARS	Reis et al., 2016 [113]
*S. officinalis* L.	herbal dust	Montenegro	subcritical water extraction	FRAP	Pavlić et al., 2016 [114]
*S. officinalis* L. subsp. *Lavandulifolia* (Vahl) Gams or Spanish sage	aerial part of plants	Spain	EOs	ORAC, DPPH, ABTS, FRAP	Cutillas et al., 2017 [115]
*S. officinalis* L.	ground leaves	Bosnia and Herzegovina	CO_2_ extract	DPPH	Pavić et al., 2019 [116]
*S. officinalis* L.	plant	Serbia	solid dispersion	DPPH	Salević et al., 2019 [117]
*S. officinalis* L.	fresh aerial parts	Italy	EO	DPPH, ABTS, FRAP, β-carotene	Tundis et al., 2020 [118]
*S. officinalis* L.	commercial-grade cosmetics	Greece	AgNPs and hydroglycolic extracts	DPPH	Siakavella et al., 2020 [119]
*S. officinalis* L. variety *Bona*	leaves	Poland	water/ethanol (50% *v*/*v*) extract	DPPH, FRAP	Francik et al., 2020 [69]
*S. officinalis* L.	dried flowers	Tunisia	aqueous extract	β-carotene, SOD, CAT, GPx	Jedidi et al., 2020 [86]
*S. officinalis* L.	leaves	Croatia	ethyl acetate	FRAP	Cvitković et al., 2021 [121]
*S. officinalis* L.	leaves	Serbia	EO	DPPH, CUPRAC, FRAP, ABTS, HRSA, TBARS	Đurović et al., 2022 [94]
*S. officinalis* L.	flowers	Tunisia	aqueous extract	ABTS, SOD, CAT, GPx	Jedidi et al., 2022 [123]
*S. officinalis* L. var *Bona*	leaves	Poland	fish oil extract	DPPH	Hrebień-Filisińska & Bartkowiak 2022 [124]
*S. officinalis* L.	commercial EO	Romania	EO	DPPH, ABTS	Mot et al., 2022 [125]

**Table 3:** Antioxidant activity of *S. officinalis* L. and other *Salvia* species from different countries.

Species	Material	Country	Extract	Antioxidant Activity Determination Method	Ref
*Salvia elegans* Vahl., *Salvia greggii* A. Gray, and *S. officinalis* L.	aerial parts (flowers, leaves, and stems)	Portugal	hexane extract	DPPH; FRAP	Pereira et al., 2018 [126]
*S. africana*, *S. officinalis ‘Icterina’*, and *S. mexicana*,	aerial parts (flowers, leaves, and stems)	Portugal	hexane extract	DPPH; TBARS; β-carotene	Afonso et al., 2019 [127]
*Salvia sclarea* and *Salvia officinalis*	inflorescences	Italy	EO	DPPH; ABTS	Ovidi et al., 2021 [92]
*S. officinalis* L., *S. virgata*, and *S. sclarea*.	aerial parts	Uzbekistan	EO	DPPH; ABTS; CUPRAC; FRAP	Gad et al., 2022 [128]
*S. hispanica* L. (Chia), in comparison with *S. officinalis* L. and *S. sclarea* L.	whole herb (leaves and stems)	Poland	methanolic extract	ABTS	Dziadek et al., 2022 [129]

## Data Availability

Not applicable.

## Abbreviations

AAPH	2,2′–Azobis(2–Amidinopropane) Dihydrochloride
ABTS	2,2′–Azinobis– (3–ethyl-benzothiazoline–6–sulfonic acid)
AgNPS	Silver Nanoparticles
CAA	Cellular Antioxidant Assay
CAT	Catalase
DCFH-DA	2′,7′–Dichlorofluorescin Diacetate
DPPH	2,2–Di(4–*tert*–octylphenyl)–1–picrylhydrazyl
FRAP	Ferric Reduction of Antioxidant Power
CUPRAC	Cupric Ion Reducing Antioxidant Capacity
DMPD	*N*,*N*–Dimethyl-*p*-phenylenediamine Dihydrochloride
EO	Essential Oil
ET	Electron Transfer
FC	Folin–Ciocalteu
FD	Freeze Drying
gGT	*gamma*–Glutamyl Transpeptidase
GR	Glutathione Reductase
GSH	Glutathione
GSHPx	Glutathione Peroxidase
GSSG	Glutathione Disulfide
GST	Glutathione–S–Transferase
HAT	Hydrogen Atom Transfer
HD	Hydrodistillation
HORAC	Hydroxyl Radical Antioxidant Capacity
LPO	Lipid Peroxidation
MAHD	Microwave–Assisted Hydrodistillation
MAE	Microwave–Assisted Extraction
MDA	Malondialdehyde
MHD	Microwave–Assisted Hydrodistillation
ORAC	Oxygen Radical Antioxidant Capacity
OxHLIA	Oxidative Hemolysis Inhibition Assay
qPCR	Quantitative PCR
ROS	Reactive Nitrogen Species
RNS	Reactive Oxygen Species
SC–CO_2_	Supercritical CO_2_ Extraction
SCWE	Subcritical Water Extraction
SD	Steam Distillation
SFE	Supercritical Fluid Extraction
SFME	Solvent–Free Microwave-Assisted Extraction
SHD	Sonohydrodistillation
SE	Soxhlet Extraction
SET	Single Electron Transfer
SLE	Solid–Liquid Extraction
SOD	Superoxide Dismutase
TAC	Total Antioxidant Capacity
TBA	Thiobarbituric Acid
TEAC	Trolox Equivalent Antioxidant Capacity
TF	Total Flavonoids
TP	Total Phenols
TPC	Total Phenolic Content
TPTZ	Tripyridyl Triazine
TRAP	Total Peroxyl Radical-Trapping Antioxidant Parameter
UAE	Ultrasound–Assisted Extraction
